# Prevalence and quantitative analysis of Epstein–Barr virus DNA and *Porphyromonas gingivalis* associated with Japanese chronic periodontitis patients

**DOI:** 10.1007/s00784-014-1387-y

**Published:** 2014-12-18

**Authors:** Ayako Kato, Kenichi Imai, Kuniyasu Ochiai, Yorimasa Ogata

**Affiliations:** 1 Department of Periodontology, Nihon University School of Dentistry at Matsudo, Chiba, 271-8587 Japan; 2 Department of Microbiology, Division of Immunology and Pathobiology, Dental Research Center, Nihon University School of Dentistry, Tokyo, 102-8310 Japan; 3 Department of Periodontology and Research Institute of Oral Science, Nihon University School of Dentistry at Matsudo, Chiba, 271-8587 Japan

**Keywords:** Chronic periodontitis, Epstein–Barr virus, Periodontopathic bacteria

## Abstract

**Objective:**

A number of studies have recently suggested Epstein–Barr virus (EBV) involvement in the pathogenesis of periodontitis. In this study, we investigated the association between major periodontopathic bacteria *Porphyromonas gingivalis* (*P. gingivalis*) and EBV in Japanese chronic periodontitis (CP) patients.

**Materials and methods:**

A group of 25 patients with CP participated in the study along with 13 individuals without periodontitis. Subgingival samples were obtained with paper points. Quantitative real-time polymerase chain reaction (PCR) was used to detect EBV DNA and *P. gingivalis*.

**Results:**

In the CP patients, EBV DNA and *P. gingivalis* were detected in both 80 % of sites with probing pocket depths (PPD) of ≥5 mm and in 40 and 36 % of sites with PPD ≤3 mm, respectively. EBV DNA and *P. gingivalis* were detected in 50 and 27 % of the sites in periodontally healthy individuals. Coexistence of EBV DNA and *P. gingivalis* was significantly higher in the deeper PPD sites of CP patients (68 %) than in the PPD sites of the healthy controls (15 %) and shallow PPD sites of CP patients (12 %). PCR-positive deeper PPD sites of CP patients for EBV DNA and *P. gingivalis* range between 3.74 × 10^3^∼2.83 × 10^9^ and 2.73 × 10^5^∼6.65 × 10^9^ (copies/ml), respectively.

**Conclusion:**

These results suggest an association between EBV DNA, *P. gingivalis*, and CP in Japanese individuals. Further studies are required to clarify this association; however, we believe that our enhanced understanding of the pathogenesis of periodontal diseases involving viral infections will lead to new treatments.

## Introduction

The pathogenesis of periodontitis is a multiple-step process involving complex interactions between specific bacterial factors, host factors, and a variety of environmental factors. Periodontitis is caused by specific periodontopathic bacteria, and it is evident that the immune responses against bacterial products and the subsequent production of proinflammatory cytokines are important for tissue destruction of the periodontium [1]. Bacterial plaque is considered to be the principal etiological factor in the onset and progression of periodontitis [2]. *Porphyromonas gingivalis* (*P. gingivalis*), *Tannerella forsythia*, and *Treponema denticola* are related to clinical parameters of chronic periodontitis, such as probing pocket depth (PPD) and bleeding on probing (BOP), and *Aggregatibacter actinomycetemcomitans* is associated with aggressive periodontitis characterized by rapid alveolar bone loss [3, 4]. In microbiological diagnosis of periodontal diseases, the subgingival plaque is commonly used to detect and quantify the bacterial species [5, 6]. Interestingly, herpes virus has been suggested to be involved in the etiology of periodontal diseases because bacterial activity does not adequately explain the clinical characteristics of periodontal diseases [7].

Epstein–Barr virus (EBV) is an enveloped herpes virus with double-stranded DNA that only infects humans. In addition, EBV is a ubiquitous virus that infects most individuals by early adulthood [8]. In primary human infection, cell-free EBV in saliva infects naive B lymphocytes, causing them to become proliferating blasts [9]. It then establishes a latent infection in those lymphocytes, which are largely nonpermissive for virus replication [10]. Among immunocompromised hosts, EBV is frequently reactivated and can induce infectious mononucleosis, autoimmune diseases, several malignancies such as Burkitt’s lymphoma, Hodgkin’s disease, nasopharyngeal carcinoma, and post-transplant lymphoproliferative disorders [8–13].

Recently, a positive association has been reported between periodontitis and EBV infection [14–16]. EBV DNA is frequently detected in saliva, salivary glands, gingival crevicular fluid, and gingival tissues [15, 17–23]. Higher levels of EBV DNA have been detected in the saliva of patients with chronic periodontitis or aggressive periodontitis [16, 17, 24]. Moreover, we demonstrated a relationship between microbial interactions and the etiology of periodontal diseases and discovered that *P. gingivalis* can induce EBV reactivation through epigenetic regulation [25].

EBV replicates at salivary glands, oral mucosal membrane, and nasopharyngeal epithelials [26, 27]. EBV DNA was detected in the throat washings of 90 % of healthy adults and in the saliva of 38 % of healthy children in Japan [27]. However, no studies have evaluated the prevalence and quantitative of EBV DNA in chronic periodontitis among the Japanese. Therefore, the purpose of this study was to determine the prevalence and quantitative analysis of EBV DNA and *P. gingivalis* among Japanese patients with chronic periodontitis.

## Material and methods

### Sampling

Thirteen periodontally healthy individuals (mean ± SD age 52.9 ± 18.0) and 25 chronic periodontitis (CP) patients (mean ± SD age 54.2 ± 13.8) were included in this study. They received dental care at Nihon University Hospital School of Dentistry at Matsudo, Japan. The Institutional Internal Review and Ethics Board at the Nihon University School of Dentistry at Matsudo approved the study (EC14-11-027-1). Written informed consent was obtained from each study subject after all procedures had been fully explained. CP patients were defined as the presence of at least two sites with probing pocket depth (PPD) ≥5 mm and attachment loss of more than 5 mm. A group of 13 individuals without periodontitis were included as healthy controls (HC). Subjects showed no clinical signs of gingival inflammation and attachment loss; moreover, no detectable bone loss was confirmed through radiographic examination and PPD ≤3 mm. All subjects were systemically healthy and had no history of periodontal treatment or any type of antibiotic therapy for at least 3 months prior to the present study. A total of 50 subgingival plaque samples were collected from two periodontal sites of PPD (≥5 and ≤3 mm) among 25 CP patients, and 26 subgingival plaque samples were collected from two sites of PPD (≤3 mm) among 13 periodontally healthy individuals. Before sampling, supragingival plaque was removed with sterile cotton pellets. Sterile paper points were then inserted to the sample site and retained for 30 s (three times). The paper points were pooled in microcentrifuge tubes and stored at −80 °C until DNA extraction.

### DNA extraction and real-time polymerase chain reaction

DNA extraction from the clinical samples was carried out using High Pure Viral Nucleic Acid Kit according to the user manual (Roche Applied Science, Mannheim, Germany). For the quantitative detection of EBV DNA and *P. gingivalis* in the samples, real-time polymerase chain reaction (PCR) was used [5, 6]. DNA extracted from the cell lines AKATA and *P. gingivalis* TDC60 were used as positive control [28, 29]. PCR was performed using the following primer sets: EBV forward, 5′-CCTGGTCATCCTTTGCCA-3′; EBV reverse, 5′-TGCTTCGTTATAGCCGTAGT-3′; *P. gingivalis* forward, 5′-AGGCAGCTTGCCATACTGCG-3′; *P. gingivalis* reverse, 5′-ACTGTTAGCAACTACCGATGT-3′; GAPDH forward, 5′-GCACCGTCAAGGCTGAGAAC-3′; and GAPDH reverse, 5′-ATGGTGGTGAAGACGCCAGT-3′, using the SYBR Premix Ex Taq in a TP800 thermal cycler dice real-time system (Takara-bio, Tokyo, Japan). PCR products comprised 95 bp for EBV, 404 bp for *P. gingivalis*, and 142 bp for GAPDH, respectively. The amplification reactions were performed in a final volume of 25 μl 2× SYBR Premix Ex Taq (12.5 μl), 0.4 μM forward and reverse primers (0.2 μl), and 50 ng complementary DNA (cDNA) (5 μl) and 10 ng cDNA (1 μl) for GAPDH. To reduce variability between replicates, PCR premixes, which contain all reagents except for cDNA, were prepared and aliquoted into 0.2-ml Hi-8-tubes (TakaRa, Tokyo, Japan). The thermal cycling conditions were 10 s at 95 °C and 40 cycles of 5 s at 95 °C and 30 s at 60 °C. Post-PCR melting curves confirmed the specificity of single-target amplification, and the expressions of EBV DNA and *P. gingivalis* relative to GAPDH were determined. The dynamic range of the real-time PCR assays were determined through serial dilution of DNA extracts either AKATA cells or *P. gingivalis* TDC60 of the standards in the range of 10^10^∼10^1^ copies per ml [28, 29].

### Statistical analysis

Fisher’s exact probability test was used to determine whether individual pathogens were associated with chronic periodontitis. *P* values <0.05 were considered statistically significant.

## Results

Characteristics of patients and clinical data are summarized in Table 1. The average PPD (*n* = 26) of the HC was 2.77 ± 0.43 mm. Among CP patients (*n* = 25), the two periodontal sites of PPD (≤3 and ≥5 mm) were 2.84 ± 0.37 and 6.28 ± 1.28 mm, respectively. BOP was detected 3.8 % in HC and 4 or 72 % in shallow (≤3 mm) or deep PPD (≥5 mm) sites from CP patients.

**Table 1:** Characteristics of subjects and clinical data in this study

	HC (13 healthy individuals)	CP (25 CP patients)
Age	52.9 ± 18.0	54.2 ± 13.8
Males	2 (15 %)	8 (32 %)
Females	11 (85 %)	17 (68 %)
PPD	2.77 ± 0.43 (*n* = 26)	2.84 ± 0.37 (≤3 mm; *n* = 25)
		6.28 ± 1.28 (≥5 mm; *n* = 25)
BOP	1 (3.8 %) (*n* = 26)	1 (4 %) (≤3 mm; *n* = 25)
		18 (72 %) (≥5 mm; *n* = 25)

*HC* healthy controls, *CP* chronic periodontitis

Table 2 describes clinical data and counts of EBV DNA and *P. gingivalis* in the CP patients. Two PCR-positive periodontal sites of PPD (≤3 and ≥5 mm) of CP patients for EBV DNA range from 4.37 × 10^4^∼9.13 × 10^6^ copies/ml (≤3 mm) and 3.74 × 10^3^∼2.83 × 10^9^ copies/ml (≥5 mm), and for *P. gingivalis* were 3.97 × 10^6^∼2.13 × 10^9^ copies/ml (≤3 mm) and 2.73 × 10^5^∼6.65 × 10^9^ copies/ml, respectively. Table 3 shows clinical data and counts of EBV DNA and *P. gingivalis* in the HC. PCR-positive sites of PPD (≤3 mm) of HC for EBV DNA range from 1.27 × 10^4^∼2.66 × 10^8^ copies/ml and for *P. gingivalis* were 4.16 × 10^6^∼6.62 × 10^9^ copies/ml, respectively.

**Table 2:** Clinical data and counts of EBV DNA and *P. gingivalis* in the chronic periodontitis patients

Subject no.	Gender	Age	PPD (≦3, mm)	BOP	EBV (copies/ml)	*P. gingivalis* (copies/ml)	PPD (≦5, mm)	BOP	EBV (copies/ml)	*P. gingivalis* (copies/ml)
1	Male	56	3	−	ND	ND	8	+	4.69 × 10^4^	7.65 × 10^5^
2	Male	57	3	−	1.09 × 10^6^	ND	8	+	7.16 × 10^4^	4.26 × 10^7^
3	Female	58	3	−	ND	2.13 × 10^9^	6	−	ND	2.86 × 10^8^
4	Female	62	3	−	1.22 × 10^6^	ND	7	+	8.85 × 10^5^	2.46 × 10^6^
5	Female	52	2	−	4.37 × 10^4^	3.97 × 10^6^	7	+	2.72 × 10^5^	2.22 × 10^6^
6	Female	40	3	−	ND	4.10 × 10^7^	6	+	ND	1.29 × 10^7^
7	Male	59	3	−	ND	ND	6	+	1.23 × 10^7^	2.79 × 10^7^
8	Female	29	3	−	9.13 × 10^6^	ND	8	+	8.60 × 10^5^	2.11 × 10^6^
9	Female	63	3	−	4.20 × 10^5^	8.95 × 10^6^	5	−	1.05 × 10^6^	1.71 × 10^7^
10	Female	27	3	−	9.33 × 10^5^	ND	5	−	3.70 × 10^7^	ND
11	Female	63	3	−	ND	7.75 × 10^7^	8	+	3.06 × 10^4^	2.21 × 10^7^
12	Male	82	3	−	4.85 × 10^6^	1.32 × 10^8^	6	+	2.83 × 10^9^	6.65 × 10^9^
13	Male	58	3	−	2.89 × 10^6^	ND	5	−	1.67 × 10^7^	2.13 × 10^8^
14	Female	49	3	−	ND	ND	6	+	3.74 × 10^3^	1.09 × 10^8^
15	Female	72	3	−	ND	ND	6	+	6.65 × 10^3^	2.79 × 10^7^
16	Female	39	2	−	ND	ND	5	+	ND	ND
17	Male	54	3	−	ND	7.81 × 10^6^	6	+	8.79 × 10^3^	3.73 × 10^6^
18	Female	42	3	−	ND	1.33 × 10^8^	10	+	4.93 × 10^4^	1.15 × 10^9^
19	Female	60	3	−	ND	ND	5	−	3.22 × 10^6^	ND
20	Female	33	3	−	ND	2.45 × 10^7^	6	+	4.35 × 10^4^	5.64 × 10^8^
21	Male	49	3	−	7.96 × 10^5^	ND	5	−	1.96 × 10^5^	ND
22	Female	61	2	−	ND	ND	6	+	ND	3.56 × 10^6^
23	Male	79	2	−	ND	ND	6	+	1.72 × 10^4^	1.64 × 10^8^
24	Female	59	3	+	1.41 × 10^5^	ND	6	+	1.21 × 10^4^	2.73 × 10^5^
25	Female	52	3	−	ND	ND	5	−	ND	ND

*ND* not detectable

**Table 3:** Clinical data and counts of EBV DNA and *P. gingivalis* in the healthy controls

Subject no.	Gender	Age	PPD (≦3, mm)	BOP	EBV (copies/ml)	*P. gingivalis* (copies/ml)	PPD, (≦3, mm)	BOP	EBV, (copies/ml)	*P. gingivalis* (copies/ml)
1	Female	64	3	−	ND	ND	3	−	ND	ND
2	Female	72	3	−	ND	ND	3	−	1.30 × 10^7^	ND
3	Female	72	3	−	ND	ND	3	−	ND	ND
4	Female	40	3	−	1.09 × 10^5^	ND	3	−	ND	ND
5	Male	28	2	−	ND	ND	3	−	ND	ND
6	Female	26	2	−	ND	ND	3	−	ND	ND
7	Female	64	3	−	8.15 × 10^7^	1.41 × 10^9^	3	−	2.58 × 10^7^	8.34 × 10^7^
8	Female	64	3	−	ND	6.62 × 10^9^	3	−	2.20 × 10^6^	ND
9	Female	46	3	−	2.66 × 10^8^	ND	2	−	3.59 × 10^7^	ND
10	Male	73	3	−	2.33 × 10^5^	ND	3	−	1.27 × 10^4^	5.96 × 10^8^
11	Female	56	2	−	5.81 × 10^5^	ND	2	+	ND	1.61 × 10^7^
12	Female	25	3	−	2.46 × 10^6^	ND	2	−	2.55 × 10^6^	ND
13	Female	58	3	−	2.56 × 10^5^	4.16 × 10^6^	3	−	ND	9.17 × 10^6^

*ND* not detectable

The occurrence frequencies of EBV DNA and *P. gingivalis* in the HC and patients with CP are listed in Table 4. EBV DNA was detected in 13 (50 %) periodontal pockets of HC and in 10 (40 %) and 20 (80 %) of the shallow (≤3 mm) and deeper PPD sites (≥5 mm) of patients with CP, respectively. *P. gingivalis* was detected in 7 (27 %) periodontal pockets of HC and in 9 (36 %) and 20 (80 %) of the shallow (≤3 mm) and deeper PPD sites (≥5 mm) of CP patients, respectively. EBV DNA and *P. gingivalis* were detected with higher frequencies in deeper PPD sites of CP patients than in PPD sites of HC. Additionally, EBV DNA and *P. gingivalis* were significantly more frequent in deeper PPD sites than in shallow PPD sites of CP patients. The occurrence frequency of EBV DNA (50 %) was higher than *P. gingivalis* (27 %) in PPD sites of HC. However, EBV DNA and *P. gingivalis* were detected at almost similar frequencies in shallow PPD sites (40 and 36 %) and in deeper PPD sites (80 and 80 %) of CP patients. Coexistence of EBV DNA and *P. gingivalis* was significantly higher in the deeper PPD sites of CP patients (68 %) than in the PPD sites of the HC (15 %) and shallow PPD sites of CP patients (12 %).

**Table 4:** Occurrence of EBV DNA and *P. gingivalis* in the subgingival samples from HC and CP patients

	Detection frequency			Significance (*P* value)		
Infectious agents	HC (*n* = 26)	CP (≤3 mm) (*n* = 25)	CP (≥5 mm) (*n* = 25)	HC vs CP (≤3 mm)	HC vs CP (≥5 mm)	CP (≤3 mm) vs CP (≥5 mm)
EBV	13 (50 %)	10 (40 %)	20 (80 %)	0.33	0.025*	0.0043**
*P. gingivalis*	7 (27 %)	9 (36 %)	20 (80 %)	0.35	0.00017**	0.0018**
EBV + *P. gingivalis*	4 (15 %)	3 (12 %)	17 (68 %)	0.52	0.00015**	0.00006**

*P < 0.05, **P < 0.01, statistically significant

## Discussion

Although bacteria play an essential role in the etiology of periodontal disease, it has become increasingly clear that herpes viruses, especially EBV, are involved in the etiology of several types of periodontal disease because bacterial activity alone does not adequately explain several clinical characteristics of periodontal disease [7, 15]. In fact, a purely bacterial cause of aggressive periodontitis does not explain why the disease tends to develop bilaterally symmetric and site-specific and why vertical bone resorption can advance at one tooth while barely affecting the periodontium of an adjacent tooth sharing the interproximal space [7, 30]. Junctional epithelial cells from the gingiva were infected with EBV, and the EBV infection was significantly increased with disease severity [31]. The results suggest that gingival epithelial cells may serve as an oral reservoir of latent EBV-infected cells. The results of this study reveal an association between EBV DNA and deep PPD sites and, likewise, *P. gingivalis* and deep PPD sites (≥5 mm) of CP lesions. Furthermore, EBV DNA and *P. gingivalis* coexist in the deep PPD sites of CP patients at high frequency (68 %). These results correlated with previous studies that showed statistically significant levels of EBV DNA in CP patients compared with healthy individuals [15, 21]. Slots and his collaborators discovered more EBV DNA in the gingival crevicular fluid and saliva of periodontal patients, and the increase in EBV counts with increasing severity of periodontitis lends substantial support to a periodontopathic role of EBV [6, 20, 22–24]. The data showed that EBV DNA was detected at ∼300-fold higher copy numbers in the PCR-positive deep PPD compared to shallow PPD sites. Moreover, *P. gingivalis* was detected at ∼3-fold higher copy numbers in the PCR-positive deep PPD compared to shallow PPD sites, in spite of having high EBV DNA and *P. gingivalis*-negative shallow PPD sites among both patients with CP and HC (Tables 2 and 3). These results suggest that high copy numbers of EBV DNA and *P. gingivalis* may reflect the severity of inflammation. In the previous report, range of counts in PCR-positive sites of periodontitis patients and periodontally normal subjects for EBV DNA (positive %; 60 and 13 %) were 2.1 × 10^3^∼8.3 × 10^8^ and 2.4 × 10^3^∼3.2 × 10^4^ copies/ml, and for *P. gingivalis* (positive %; 87 and 13 %) were 5 × 10^3^∼1 × 10^10^ and 2.1 × 10^4^∼3.1 × 10^6^ copies/ml [6]. The results showed that copy numbers of EBV DNA and *P. gingivalis* in the periodontal lesions were almost the same as in our study (Table 2); however, their copy numbers in the normal subjects were lower compared to our data (Table 3). EBV DNA was detected at higher rate in the PPD sites of HC (50 %) than in the shallow PPD sites of CP (40 %). On the other hand, *P. gingivalis* was detected at higher rate in the shallow PPD sites of CP (36 %) than in the PPD sites of HC (27 %) (Table 4). These results might be caused by higher latent infection rate of healthy Japanese by EBV.

We have previously reported B cell marker CD19 immunostaining showed that a large number of B cells had infiltrated into the gingival connective tissues of patients with periodontitis [15]. And the results of in situ hybridization using EBV-encoded small RNA (EBRE) showed a large number of B cells in the same location that were EBER-positive [15]. Results suggest that EBV copy numbers in the subgingival plaque samples may relate to the severity of inflammation and the numbers of inflammatory cell infiltration in the gingiva.

The mechanisms of EBV reactivation and activated EBV progressing to periodontal disease have not been determined. Latent EBV in B cells can be reactivated to switch to lytic replication. EBV reactivation can be induced in vitro by a variety of stimuli, including 12-*O*-tetradecanoylphorbol-13-acetate and anti-immunoglobulin, but a causal relationship between a coinfection with EBV and periodontopathic bacteria and the disruption of viral latency is not well understood. We have previously reported that the culture supernatant from *P. gingivalis*, which contains high concentrations of butyric acid, inhibits histone deacetylase and thus increases histone acetylation and transcriptional activity of the EBV BZLF1 gene, which encodes the master regulator protein (ZEBRA) for the transition from latency to the lytic replication cycle [25]. Given that regulation of the switch from latency to reactivation is an initial key step in EBV infection, these observations suggest that butyric acid-producing periodontopathic bacteria, such as *P. gingivalis*, have the potential to trigger EBV reactivation in the oral cavity of infected individuals [25]. EBV-infected inflamed periodontal sites tend to harbor elevated levels of periodontopathic bacteria [15, 21, 23, 32]. Furthermore, bacterial and viral coinfections were also reported more frequently in deep periodontal pockets [14, 15, 21, 23]. EBV-1, EBV-2, and *P. gingivalis* were detected in 72.5, 10, and 95 % of sites with probing pocket depths ≥6 mm, respectively [21]. We also reported that EBV and *P. gingivalis* were detected in 66 and 65 % of sites with probing pocket depths ≥5 mm, and EBV DNA and *P. gingivalis* coinfection was found in 42 % of sites with probing pocket depths ≥5 mm [15]. These observations suggest that a “negative chain reaction” by EBV and periodontopathic bacteria may contribute to the etiopathogenesis of periodontitis [33].

In summary, we performed quantitative analysis of EBV DNA and *P. gingivalis* in Japanese chronic periodontitis patients which to our knowledge is the first such attempt. EBV DNA and *P. gingivalis* were detected in higher copy numbers in PCR-positive deep PPD and showed a higher incidence of the coexistence as compared to shallow PPD sites. Taking into account that periodontopathic anaerobic bacteria may increase the virulence of EBV via reactivation of EBV through butyric acid, their suppression or eradication may become an effective treatment to block EBV reactivation for early treatment or prevention of chronic periodontitis.
